# Accurate diagnosis and effective treatment of abnormal meridians in erectile dysfunction patients based on infrared thermography: an electrophysiological technique study

**DOI:** 10.1038/s41443-024-00859-w

**Published:** 2024-03-20

**Authors:** Wang Zihao, Liu Kaifeng, Zhang Shengmin, Gong Yongzhan, Lu Pengjie

**Affiliations:** 1 The Yangzhou School of Clinical Medicine of Dalian Medical University, Yangzhou, China; 2 Northern Jiangsu People’s Hospital of Jiangsu Province, Yangzhou, China; 3 Northern Jiangsu People’s Hospital Affiliated to Yangzhou University, Yangzhou, China; 4 The Yangzhou Clinical Medical College of Xuzhou Medical University, Yangzhou, China

**Keywords:** Erectile dysfunction, Clinical trials

## Abstract

**Abstract:**

An increasing body of research has demonstrated that appropriate stimulation of the meridians and acupoints in the human body can play a preventative and therapeutic role in diseases. This study combines the use of infrared thermography with intelligent electrophysiological diagnostic system (iEDS) to accurately diagnose and apply transdermal low-frequency electrical stimulation to treat abnormal meridians in patients with erectile dysfunction (ED). The treatment protocol included 6 treatments (each lasting 30 min and performed twice a week). The International Index of Erectile Function-5 (IIEF-5), Patient Health Questionnaire-9 (PHQ-9), Generalized Anxiety Disorder-7 (GAD-7), and Erection Hardness Scale were used to assess treatment results. A total of 62 patients were included in this study, with 31 patients in the treatment group and 31 patients in the sham therapy group. After six treatments, the treatment group improved significantly in IIEF-5 (15.52 ± 2.06 vs. 18.84 ± 2.67, *p* < 0.001), PHQ-9 (8.32 ± 6.33 vs. 4.87 ± 4.41, *p* < 0.001), GAD-7 (5.32 ± 5.08 vs. 2.94 ± 3.31, *p* = 0.003), and EHS (2.48 (2.00, 3.00) vs. 2.90 (2.00, 3.00), *p* = 0.007). After six sham treatment sessions, no improvements in any of the scores were reported in the sham therapy group. Following that, this group had an additional six treatments of regular therapy, which resulted in statistically significant improvements in IIEF-5 (16.65 ± 1.96 VS. 19.16 ± 2.40*, p* < 0.001), PHQ-9 (8.81 ± 6.25 VS. 4.97 ± 4.36, *p* < 0.001), GAD-7 (5.74 ± 5.18 VS. 3.68 ± 3.42, *p* < 0.001), and EHS (2.61 (2.00, 3.00) VS. 3.03 (2.00, 4.00), *p* = 0.003). No adverse events were reported regarding penile discomfort, pain, injury, or deformity.

**Clinical trials:**

The study protocol is registered in the Clinical Trials Registry with the identification number ChiCTR2300070262.

## Introduction

A great number of studies in recent years have shown that stimulating specific acupoints can effectively treat a wide range of disorders, including Parkinson’s disease [1], polycystic ovarian syndrome [2], knee osteoarthritis [3], and migraine [4]. In traditional Chinese medicine theory, there exists a mutual dependence between qi and blood. Qi is one of the fundamental substances that constitute and sustain human body and its life activities. It propels and regulates the vital functions of the human body. When the flow of Qi ceases within the body, it signifies the termination of life. Blood serves as the carrier of Qi, as Qi must rely on blood in order to ensure its proper functioning. Meridians and acupoints act as conduits for the flow, transit, and distribution of qi and blood throughout the body [5]. Preventive and therapeutic benefits in the treatment of associated disorders can be accomplished by correctly activating these pathways [6]. Acupoints can be stimulated with numerous types of energy, including mechanical stimulation by acupuncture and massage [7], heat stimulation by moxibustion [8], and electrical, magnetic, and laser stimulation. We used transcutaneous low-frequency electrical stimulation in concert with meridians and acupoints in our study to mimic the effects of acupuncture while avoiding the unpleasantness associated with needle insertion, such as pain and bleeding. This therapy achieves outcomes comparable to conventional acupuncture and electroacupuncture while displaying increased acceptance [9].

Meridians are an exceedingly ancient technique; however, nowadays, an increasing number of contemporary methodologies such as Laser Doppler blood flow monitoring, near infrared spectroscopy, and infrared thermography (IRT) are being applied in the study of meridians. IRT is the result of combining infrared energy camera technology with computer multimedia technology. The thermal field of the human body can be recorded with this imaging method. IRT provides visibility and non-invasiveness by dynamically detecting variations in body surface temperature. As a result, it is widely used in traditional Chinese medicine research [10].

Previous research on meridians employing IRT have generally focused on illness screening, detection, progression tracking, and therapy result verification [11]. However, there has been little in-depth study on illness diagnosis, particularly the identification and therapy of aberrant meridians in the human body. In this paper, we provide a unique method for combining infrared thermography with intelligent electrophysiological diagnostic system (iEDS). In this study, we utilized the theories of traditional chinese medicine on meridians to identify abnormal meridians in patients with erectile dysfunction (ED) through monitoring of target organs, target areas, and the overall condition. Additionally, we simultaneously determined the parameters for electro-physiological treatment. One of the most common male sexual dysfunctions is ED, which refers to the penis’s inability to generate or sustain a sufficiently hard erection for successful sexual intercourse [12]. It has a tremendous influence on the quality of life of both patients and their spouses, as well as the stability of their relationships [13]. Furthermore, ED serves as an early signal of a variety of chronic ailments, including cardiovascular/cerebrovascular diseases, metabolic abnormalities, and psychological issues [14, 15]. phosphodiesterase type 5 (PDE5) inhibitors are the most often used therapeutic medicines for ED therapy [16]. However, study data show that PDE5 inhibitors are ineffective in roughly 35% of ED patients [17].

In this study, we used iEDS to administer electrical stimulation to ED patients with a variety of parameter settings. Concurrently, real-time infrared thermography was used to monitor and record temperature fluctuations in the target organ area. By comparing temperature changes of the target organ area, effective electrical stimulation parameters were determined. The goal of our study is to find the best electrical stimulation parameters and combinations for treating patients by comparing temperature changes in target organ areas before and after electrical stimulation with different parameters, and subsequently evaluate the therapeutic effects.

## Materials and methods

### Research objective

The followings were the patients’ inclusion criteria: (1) Patients with IIEF-5 scores less than 22, (2) Patients who are in good physical condition, maintain regular daily routines, have a stable sexual partner, and can engage in normal sexual activity from the beginning to the end of the study, (3) Patients who have not taken any medication for ED in the 4 weeks prior to participating in this study and have not undergone any physical or surgical treatments for ED within the past 6 months, (4) Patients with normal results in their reproductive hormone tests. The followings were the patients’ exclusion criteria: (1) Patients with significant organic abnormalities affecting penile erection, (2) Patients with severe cardiovascular diseases that prevent sexual activity, (3) Patients who have undergone pacemaker surgery, (4) Patients with a history of neurological or psychiatric disorders, and (5) Patients with a history of substance abuse. Before the start of the study, patients completed IIEF-5, the Patient Health Questionnaire-9 (PHQ-9), the Generalized Anxiety Disorder-7 (GAD-7), and the Erection Hardness Score (EHS) under the guidance of physicians. These questionnaires were completed again during the follow-up visit two weeks after the completion of treatment. All participants in the study had previously signed informed consent forms. The research protocol has been registered with the Clinical Trial Registration Center under the identification code ChiCTR2300070262.

### Preparation for inspection

The examination room is free of direct sunlight and air convection, and the temperature and humidity levels are carefully controlled at 22–26 °C and 40–60%, respectively. Patients must refrain from using vasodilating and vasoconstricting drugs for at least 24 h before the evaluation. Furthermore, patients should not eat or drink for at least 4 h before the test and should avoid being exposed to very hot or cold settings within 1 h before the evaluation. Patients should empty their bladders 15 min before the test, relax their belts, remove watches, necklaces, and other jewelry, and spend 10 min soothing themselves. They are allowed to stand or sit on a hard chair, but not on soft chairs such as couches.

### Check for abnormal meridians

**Fig. 1:**
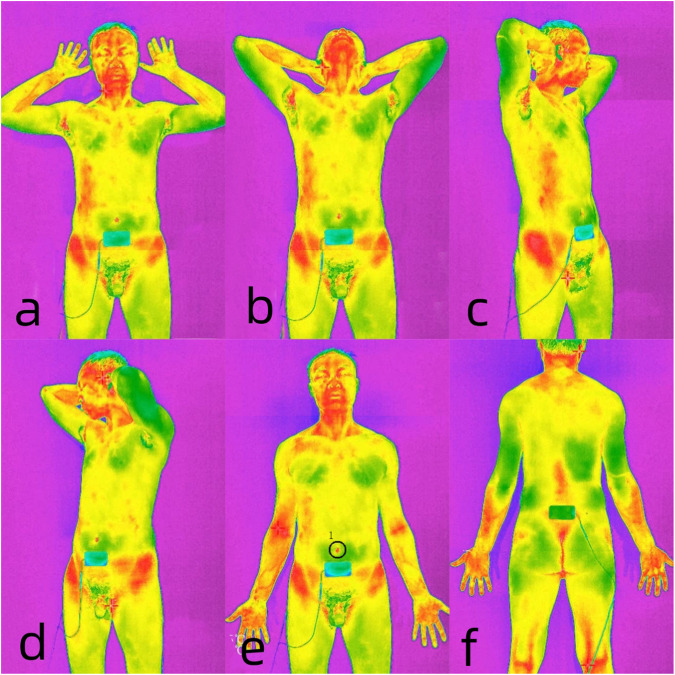
The six fundamental maneuvers are as follows. **a** Elevate the hands in close proximity to the ears, with palms oriented forward and all five fingers extended. **b** Position the hands on the head, tilting the head backwards to expose the neck. **c** Place the hands on the head and pivot 45° to the left. **d** Place the hands on the head and pivot 45° to the right. **e** Assume a stance with legs slightly apart, lowering the hands to the same width as the shoulders, with palms facing forward and all five fingers extended. **f** Maintain the fifth posture and rotate 180° in a backward direction.

**Fig. 2:**
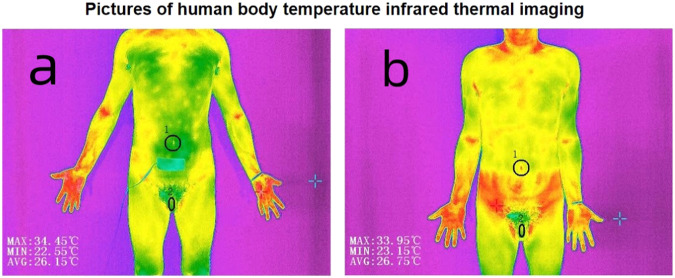
Left: infrared images before treatment. Right: infrared images after treatment.

The patient undresses completely and places two electrodes on the Guanyuan (CV4) and Mingmen (GV4). Then, patient enter the medical infrared thermography inspection cabin. The patient is instructed to perform six movements (Fig. 1), with each movement being captured as a thermal image to serve as a baseline for infrared imaging photographs. The electrodes were linked to the BioStim pro (Foshan Shanshan Datang Medical Technology Co., Ltd., FoShan, China), and the 12 built-in electrical stimulation parameters were successively applied to the patient (Table 1). Each parameter was stimulated for 30 s, and an infrared thermographic picture was acquired to compare and evaluate with the baseline image (Fig. 2).

**Table 1:** Electrical stimulation parameters and Position of electrode sheets.

Meridian^▲^	Electrical stimulation parameters	Position of electrode sheets
Kidney Meridian (KI)	18 Hz/300 μs	Taixi (KI3), Yingu (KI10), Henggu (KI11), Dahe (KI12)
Spleen Meridian (SP)	28 Hz/300 μs	Sanyinjiao (SP6), Jimen (SP11)
Governor vessel (GV)	82 Hz/300 μs	Yaoyangguan (GV3), Mingmen (GV4)
Sanjiao Meridian (SJ)	89 Hz/300 μs	Yangchi (SJ4), Sanyangluo (SJ8)
Conception vessel (CV)	86 Hz/300 μs	Qugu (CV2), Zhongji (CV3), Guanyuan (CV4), Shimen (CV5), Qihai (CV6)
Bladder Meridian (BL)	69 Hz/300 μs	Fengmen (BL12), Shenyu (BL23), Shangliao (BL31), Huiyang (BL35), Zhishi (BL52)
Gallbladder Meridian (GB)	58 Hz/300 μs	Jingmen (GB25), Daimai (GB26), Xuanzhong (GB39)
Stomach Meridian (ST)	47 Hz/300 μs	Daju (ST27), Qichong (ST30), Zusanli (ST36)
liver Meridian (LR)	9 Hz/300 μs	Ligou (LR5), Zhongdu (LR6), Zhangmen (LR13)
Small Intestine Meridian (SI)	98 Hz/300 μs	Yanggu (SI5)
Lung, Heart, Pericardium Meridian (LU,HT,PC)	36 Hz/300 μs	Lieque (LU7), Shaofu (HT8), Neiguan (PC6)
Large Intestine Meridian (LI)	80 Hz/300 μs	Hegu (LI4), Pianli (LI6)

Note:▲Arrange the meridians in descending order based on the frequency of occurrence of abnormalities.

### Determination of effective parameters and treatment

**Fig. 3:**
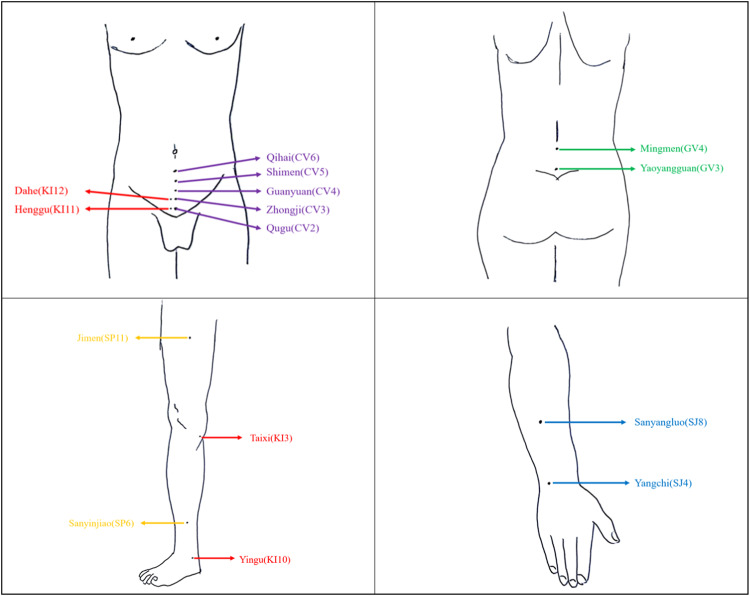
The five most common abnormal meridians in patients with ED in this study. KI Kidney Meridian, CV Conception vessel, GV Governor vessel, SP Spleen Meridian, SJ Sanjiao Meridian.

The value that caused a temperature shift more than 0.5 °C in the umbilicus area in the shortest amount of time was considered the effective parameter during the electrical stimulation procedure. After determining the appropriate parameters, the electrode pads are applied to the corresponding areas (Table 1) (The five most frequently observed abnormal meridians in our study, as well as the locations for electrode placement, are depicted in Fig. 3), and then connected to the BioStim ble6 (Foshan Shanshan Datang Medical Technology Co., Ltd., FoShan, China). Adjust the magnitude of the current to allow patients to perceive electrical stimulation without experiencing excessive pain. Each aberrant meridian was subjected to 30 min of electrical stimulation twice a week for a total of six therapy sessions.

Between November 2022 and August 2023, A total of 62 patients with ED were included and evenly divided into two groups using complete random allocation. The group 1 (treatment group) had the detected aberrant meridians electrically stimulated. The group 2 (sham therapy group) got electrical stimulation treatments on normal upper limb meridians, which had no therapeutic impact on ED patients since they were normal meridians. After conducting the sham treatment, proceed with administering the standard treatment to the second group of patients. This study was a single-blind, randomized placebo-controlled trial. Patients remained unaware of their group assignment until the completion of the treatment.

### Clinical outcome measurements

The IIEF-5 is a commonly employed questionnaire for assessing symptoms in individuals diagnosed with ED. The questionnaire has five questions, with four assessing the degree of erection and one assessing satisfaction with sexual intercourse. The total score ranges from 0 to 25, with a score below 22 indicating the presence of ED symptoms [18, 19]. There is an interaction between ED and the psychological status of patients [20]. Research suggests that while psychological state may not be the direct cause of ED, it can potentially induce anxiety and depressive symptoms in patients [21]. We used PHQ-9 [22, 23] and GAD-7 [24, 25] to assess the patients’ psychological health. Furthermore, EHS [26] was used to quantify the quality of penile erection on a scale of 0 to 4.

### Statistical methods

IBM SPSS Statistics 22.0(SPSS Inc., Chicago, IL, USA) was used for statistical analysis. The single-sample K-S test is employed to examine the normality of the data. Data exhibiting normal distribution is presented as mean ± standard error and is compared using paired-sample *t*-test；Data that does not conform to normal distribution is represented as median (first quartile, third quartile) and is compared using Wilcoxon signed-rank test. *P* < 0.05 was considered statistically significant.

## Results

### Patient’s baseline

A total of 62 patients participated in our study, including 31 patients in the treatment group and 31 patients in the sham therapy group. Prior to treatment, there were no significant differences between the two groups in terms of age, BMI, IIEF-5, GAD-7, PHQ-9, and EHS (Table 2).

**Table 2:** Comparison of basic characteristics between the two groups before treatment.

	Age^a^	BMI^a^	IIEF-5^a^	PHQ-9^a^	GAD-7^a^	EHS^b^
Group 1	31.06 ± 6.15	25.28 ± 3.59	15.52 ± 2.06	8.32 ± 6.33	5.32 ± 5.08	2.48 (2.00, 3.00)
Group 2	30.85 ± 5.26	27.02 ± 4.34	15.71 ± 1.85	8.81 ± 6.25	5.74 ± 5.18	2.61 (2.00, 3.00)
*P*	0.36	0.71	0.70	0.52	0.96	0.45

Note: *IIEF-5* International Index of Erectile Function-5, *PHQ-9* Patient Health Questionnaire-9, *GAD-7* Generalized Anxiety Disorder-7, *EHS* Erection Hardness Score.

^a^The data follows a normal distribution, thus the the paired sample *t*-test is utilized.

^b^The data follows a non-normal distribution, thus the Wilcoxon signed-rank test is utilized.

### Post-treatment efficacy

In group 1 (treatment group), after receiving treatment, patients’ IIEF-5 scores improved from 15.52 ± 2.06 to 18.84 ± 2.67, *p* < 0.001. PHQ-9 scores decreased from 8.32 ± 6.33 to 4.87 ± 4.41, *p* < 0.001. GAD-7 scores decreased from 5.32 ± 5.08 to 2.94 ± 3.31, *p* = 0.003. EHS score improved from 2.48 (2.00, 3.00) to 2.90 (2.00, 3.00), *p* = 0.007 (Table 3). In group 2 (sham therapy group), there was no statistically significant improvement in any of the scores after sham treatment. After this, these patients underwent normal treatments and the patients’ IIEF-5 scores improved from 16.65 ± 1.96 to 19.16 ± 2.40, *p* < 0.001. PHQ-9 scores decreased from 8.81 ± 6.25 to 4.97 ± 4.36, *p* < 0.001. GAD-7 scores decreased from 5.74 ± 5.18 to 3.68 ± 3.42, *p* < 0.001. EHS score improved from 2.61 (2.00, 3.00) to 3.03 (2.00, 4.00), *p* = 0.003 (Table 4). During both the therapy phase and the follow-up period, no patients reported any penile discomfort, pain, damage, or deformity.

**Table 3:** Clinical outcomes of pre- and post-electrophysiologic treatment in group 1.

	Before treatmen	After treatmen	*P*
IIEF-5^a^	15.52 ± 2.06	18.84 ± 2.67	< 0.001
PHQ-9^a^	8.32 ± 6.33	4.87 ± 4.41	< 0.001
GAD-7^a^	5.32 ± 5.08	2.94 ± 3.31	= 0.003
EHS^b^	2.48 (2.00, 3.00)	2.90 (2.00, 3.00)	= 0.007

Note: *IIEF-5* International Index of Erectile Function-5, *PHQ-9* Patient Health Questionnaire-9, *GAD-7* Generalized Anxiety Disorder-7, *EHS* Erection Hardness Score.

^a^The data follows a normal distribution, thus the the paired sample *t*-test is utilized.

^b^The data follows a non-normal distribution, thus the Wilcoxon signed-rank test is utilized.

**Table 4:** Clinical outcomes of pre- and post-electrophysiologic treatment in group 2.

	Before treatmen	After placebo	*P*^▲^	After treatmen	*P*^▲^
IIEF-5^a^	15.52 ± 2.06	16.26 ± 1.81	0.231	19.16 ± 2.40	< 0.001
PHQ-9^a^	8.32 ± 6.33	8.77 ± 6.73	0.915	4.97 ± 4.36	< 0.001
GAD-7^a^	5.32 ± 5.08	6.10 ± 5.69	0.133	3.68 ± 3.42	< 0.001
EHS^b^	2.48 (2.00, 3.00)	2.55 (2.00, 3.00)	0.564	3.03 (3.00, 4.00)	0.003

Note: *IIEF-5* International Index of Erectile Function-5, *PHQ-9* Patient Health Questionnaire-9, *GAD-7* Generalized Anxiety Disorder-7, EHS Erection Hardness Score.

^a^The data follows a normal distribution, thus the the paired sample *t*-test is utilized.

^b^The data follows a non-normal distribution, thus the Wilcoxon signed-rank test is utilized.

^▲^Compared to the data before treatment.

## Discussion

Low-frequency currents are pulsed currents with frequencies less than 1000 Hz in the medical profession, and low-frequency electrophysiology technology has found widespread application in clinical practice [27].The majority of biochemical activities in the human body involve the production and dissipation of heat, with heat loss largely occurring through cutaneous blood flow. When sickness develops, changes in general or local heat balance occur, resulting in temperature differences in the relevant locations. The importance of these variations in body surface temperature for illness diagnosis and therapy recommendations is now being investigated by researchers [28]. Notably, there is a connection between specific body parts’ surface temperatures with meridian channels, and acupoints, these places have greater temperatures than other areas [29]. The temperature of meridians shows whether the relevant functional areas are in a normal or pathological state [30]. Due to its sensitivity to infrared radiation and body surface temperature, IRT is a technology that is being used more and more in clinical research [31]. However, prior research has mostly focused on examining the connections between certain meridians and organs as well as making the distinction between meridian and non-meridian areas. Our study utilized IRT to visualize the data and combine them with the Chinese medicine meridian theory. We wanted to promote tailored diagnostic and treatment methods, ultimately resulting in increased clinical efficacy, by taking into account the diverse bodily constitution among individuals.

Bioelectrical signals are found throughout the human body’s physiological and pathological processes [32]. These electrical signals regulate several activities inside the organism, including the heart, skeletal muscles, skin, and neurological system. They are involved in functions such as heartbeat, bone regeneration, muscular contraction, wound healing, and neural transmission [33]. Electrical stimulations are critical to regulating cell proliferation, differentiation, adhesion, migration, and extracellular matrix production [34]. Electrical stimulation affects cellular depolarization, which has an effect on the organism. Low-frequency pulse currents of sufficient strength can create enough charge to activate neurons and cause an action potential. For cellular depolarization, different organs have set electrical stimulation parameters [35]. Despite the fact that the goal of treatment is the same, various tissue structures need varied electrical stimulation parameters [36]. The stimulation pulse width and waveform must also be addressed [37]. Our study’s goal was to place electrode patches to relevant acupoints of different meridians and use electrical stimulation parameters that corresponded to the meridians to get a batter therapeutic impact.

We placed electrode patches on the Guanyuan and Mingmen acupoints during the examination. First of all, the Spleen Meridian, Kidney Meridian, Liver Meridian, and Conception Vessel all converge in Guanyuan. Mingmen, an acupoint on the Governor Vessel, which is referred to as the “sea of yang qi” where all six yang meridians meet, is situated between the two kidneys. Second, both of these acupoints are situated in the lower dantian, which is shielded by adipose and muscular tissues and is far from the heart and brain. Therefore, we can effectively stimulate the meridians while reducing any discomfort that patients may feel by providing electrical stimulation with varying parameters. ED is known as “impotence” in traditional Chinese medicine. It refers to adult men’ failure to acquire or sustain a hard erection during sexual intercourse while not having reached the age-related loss in sexual function. Acupuncture treatment for ED focuses mostly on the Kidney Meridian, the Governor Vessel, the Spleen Meridian, and the Conception Vessel, which is consistent with our results. Furthermore, we discovered a considerable presence of the Sanjiao Meridian in the therapy of ED, which necessitates further in-depth investigation to explore its underlying causes.

Based on our previous experiences, most patients can accept non-pharmacological treatments to improve ED symptoms. However, frequent hospital visits and long waiting times for treatment are the main reasons for their treatment abandonment. In order to address these concerns, we have made improvements to the previous treatment approach. First, we accurately identify the abnormal meridians in patients. and then, simultaneous electrophysiological treatment is administered to all identified abnormal meridians, effectively reducing patient waiting and treatment times. Moreover, multiple courses of treatment can be provided to the patients.

However, our study does have some limitations. First, although we strictly adhered to the inclusion and exclusion criteria in patient selection, we did not exclude psychogenic erectile dysfunction (PED), which may have had a placebo effect on the results. Second, the skin temperature of the human body is influenced by physiological, pathological, and environmental factors. Physiological factors mainly include circadian rhythms [38], skin color [39], subcutaneous fat [40], and metabolic rate. Pathological factors primarily result from specific disease conditions. Environmental factors mainly encompass ambient temperature and relative humidity [41]. In this study, we rigorously controlled the room temperature and humidity, providing sufficient time for patients to acclimate to the environment. All these measures effectively reduced data inaccuracy and ensured the reliability of the results. However, both IRT and human body temperature are influenced by numerous factors. Although we have implemented various measures to control some of these factors, it is inevitable that other unknown factors may still introduce interference. In this study, although there was a statistically significant improvement in IIEF-5 score, the magnitude of improvement was not substantial. Therefore, further observation of the impact of multiple treatment courses on the improvement of ED symptoms will be conducted in our subsequent research.

There have been numerous discussions regarding the mechanisms by how electrical stimulation improves ED. The mainstream mechanisms include the improvement of corpus cavernosum smooth muscle structure, increased intracorneal pressure, promotion of endothelial cell release of nitric oxide (NO) [42], and facilitation of smooth muscle and neural regeneration within the corpus cavernosum [43]. In our study, each group was comprised of only 31 patients, and we just investigated the therapeutic effects of transcutaneous low-frequency electrical stimulation of meridians on ED. In the next step, we can continue to expand the sample size and incorporate combination therapy with medication to further explore the methods, mechanisms, and principles of using electrical stimulation through the meridians for the treatment of ED.

## Conclusions

An increasing number of studies indicate that appropriate stimulation of the human meridians and acupoints can play a role in disease prevention and treatment. Our study entailed administering low-frequency electrical stimulation to patients with varying parameters, in conjunction with employing a medical infrared thermal imager to observe and record the patients’ surface temperature. By comparing the changes in temperature, it is possible to accurately identify abnormal meridians in the body and determine suitable treatment plans for the patients. The results demonstrate a significant improvement in the patients’ IIEF-5, PHQ-9, GAD-7, and ESH scores after treatment, providing evidence of the effectiveness of our intervention. Moving forward, our next steps will involve expanding the sample size and exploring the mechanisms of meridian therapy for treating ED.

## Data Availability

The data that support the findings of this study are available from the corresponding author upon reasonable request.
